# Fragment Ion Abundance Reveals Information about Structure and Charge Localization in Highly Charged Proteins

**DOI:** 10.1021/jasms.3c00196

**Published:** 2023-07-21

**Authors:** Thomas A. Shoff, Ryan R. Julian

**Affiliations:** Department of Chemistry, University of California, Riverside, California 92521, United States

**Keywords:** native mass spectrometry, top-down mass spectrometry, collisional activation, charge localization, electron-transfer dissociation

## Abstract

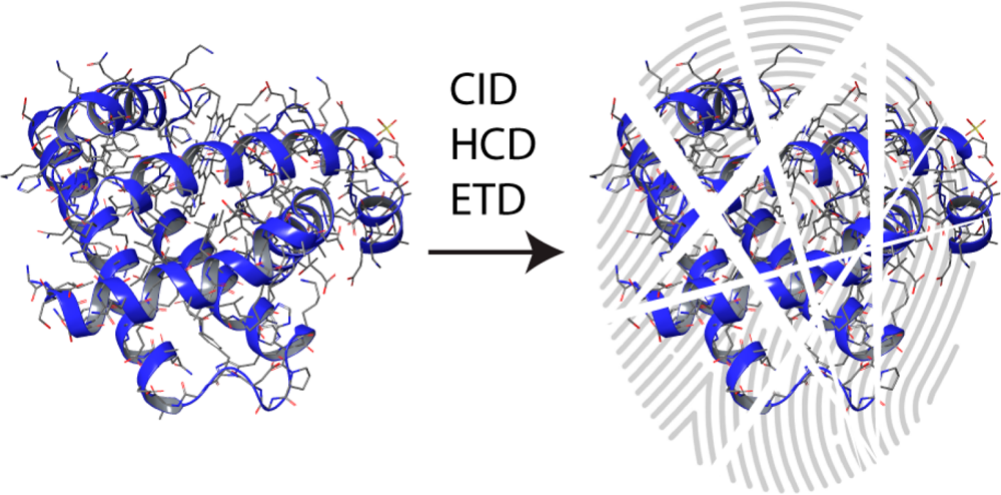

Top-down mass spectrometry (MS) is a versatile tool that has been employed to investigate both protein sequence and structure. Although a variety of different fragmentation methods are available in top-down MS that can potentially yield structural information, quantifying differences between spectra remains challenging. Herein, we show that subtle differences in spectra produced by a variety of fragmentation methods are surprisingly sensitive to protein structure and/or charge localization, even in highly unfolded proteins observed in high charge states. In addition to exposing information about the protein structure, differences in fragmentation also reveal insight into the mechanisms underlying the dissociation methods themselves. The results further reveal that small changes in experimental parameters (such as the addition of methanol instead of acetonitrile) lead to changes in structure that are reflected in statistically reproducible differences in dissociation. Collisional annealing of structurally dissimilar ions in the gas phase eventually leads to dissociation spectra that are indistinguishable, suggesting that structural differences can be erased by sufficient thermal activation. Additional experiments illustrate that identical charge states of the same protein can be distinguished if those produced directly by electrospray are compared to ions manipulated by *in vacuo* proton-transfer charge reduction. Overall, the results show that subtle differences in both three-dimensional structure and charge-site localization can influence the abundance of fragment ions produced by top-down MS, including dissociation methods not typically thought to be structurally sensitive.

## Introduction

Proteins are important molecular machinery in biology that typically function by adopting specific structures. Elucidating protein structures is important for complete understanding of biological systems, and mass spectrometry (MS) has emerged as a useful technique for investigating important aspects of protein structure in both solution and the gas phase due to its inherent speed and sensitivity.^1−12^ For studies focused on the gas-phase approach, factors that influence protein structure *in vacuo* have been the subject of considerable discussion. It is unlikely that the lowest energy structures for a protein in the gas phase are similar to those in aqueous solutions, but results derived from numerous studies suggest that under appropriately gentle ionization conditions (collectively coined native-MS), aspects of native structures can be retained in the gas phase.^13−18^ In contrast, experiments conducted under more denaturing conditions produce proteins in high charge states that are largely unfolded and therefore not native-like in terms of tertiary structure.^8,19−21^ It is unclear in such experiments whether any connections to the native structure remain or if the elongated structures so produced are able to adopt the lowest energy structures for the gas-phase environment.

In the complete absence of solvent, the number and localization of charged sites will strongly influence the properties and behavior of proteins in the gas phase.^22−24^ In most cases (even for high charge states), the number of basic sites exceeds the number of excess protons, leading to the existence of many potential “protomers”, i.e., isomers that only differ by where protons are located. Additionally, for proteins, the number of charge sites most likely exceeds the number of excess protons because proteins have a strong preference to form zwitterionic pairs where some acidic sites are deprotonated.^25,26^ These zwitterionic pairs do not increase the charge state of a protein but can greatly increase the number of potential protomers. In the context of the three-dimensional protein structure, the relationship between the protomeric state and structure is not immediately obvious. It is possible that protomers with similar 3D structures could exist, differing primarily in the specific sites of protonation or deprotonation. It is also feasible that significantly different three-dimensional structures could adopt the same protomeric state.

One method for examining structure in the gas phase is to dissociate the ionized protein and extract structural information based on the fragment-ion abundance. There are a variety of methods capable of fragmenting intact proteins, each with potential strengths and weaknesses in terms of structural interrogation. Dissociation based on electron capture or transfer (ECD/ETD) yields high sequence coverage and does not require heating of the ion to initiate dissociation.^27−30^ Ultraviolet photodissociation (UVPD) is also useful for protein characterization, yielding high sequence coverage through a variety of dissociation mechanisms (including some that heat the protein).^31−35^ Collisional activation can also be used with intact proteins by way of many low energy collisions (as occurs in an ion trap) or by fewer but higher energy collisions (as occurs in beam-type arrangements). Although it might be reasonable to expect that the heating process preceding dissociation by collisional activation might erase any memory of solution phase structure, recent results obtained by the Loo lab suggest otherwise.^36^ They were able to use higher-energy collisional dissociation (HCD) to obtain structural information about protein complexes under native conditions. Collisional activation can also be coupled with radical-directed dissociation (RDD) to explore protein structure. In these experiments, photocleavage of labile bonds creates a radical at a specific site, which then facilitates fragmentation in its immediate vicinity. The combination of initial radical location and final dissociation points can then serve as proximity constraints for modeling potential structures.^37^ To interpret the data obtained in any of these dissociation-based experiments, the presence or absence of certain fragment ions is often attributed to specific structural features. Although less commonly used, differences in the abundance of fragment ions between two systems have also been interpreted as the result of structural dissimilarities.^31,32,35,38−42^

In this work, we utilize a statistical framework to analyze differences in the fractional abundance of fragment ions common to two top-down mass spectra acquired with a variety of proteins in high charge states. We explore data produced under a variety of alternate conditions including differences in electrospray solution composition, the extent of gas-phase annealing, and charge state modulation after ion–ion reactions. The alternate conditions are explored with ion-trap collision-induced dissociation (CID), HCD, and ETD. The results are discussed in relation to the three-dimensional protein structure, charge localization, and mechanisms underlying the fragmentation methods. It is revealed that a surprising extent of structural variety exists in the gas phase for highly charged proteins and that all fragmentation methods are sensitive to this structural diversity to some extent.

## Experimental Section

### Materials

All reagents and proteins were used without purification. Cytochrome *c* (equine), myoglobin (equine), and hemoglobin (human) were purchased from Sigma-Aldrich (St. Louis, MO). Organic solvents were purchased from Fisher Scientific.

### Sample Preparation

All solutions were prepared with 10 μM protein dissolved in varying amounts of water (H_2_O), methanol (MeOH), acetonitrile (ACN), and formic acid (FA) as described in the text.

### Mass Spectrometry

All experiments were performed on a Thermo Orbitrap Fusion Lumos instrument. Proteins were introduced into the instrument via nanospray using a nano flex source from Thermo Scientific that was modified with a platinum wire to allow the use of tips pulled from borosilicate glass (Harvard Apparatus GC100T-10). Nanospray tips were ∼1–15 μm in diameter and had a taper length of ∼1 mm. Proteins were isolated using the quadrupole and subjected to CID, HCD, or ETD prior to analysis in an Orbitrap mass analyzer. In subthreshold CID experiments, CID energy was incrementally increased until just below the observation of fragment ions, and the protein ion was then reisolated and subjected to MS^3^ fragmentation by CID, HCD, or ETD. For proton transfer charge reduction experiments (PTCR), nitrogen-adducted fluoranthene (*m*/*z* 216 Da) was used as a proton-scavenging anion.^43^ Following proton transfer during MS^2^, the desired charge state was isolated and subjected to MS^3^ fragmentation. All mass spectra were acquired in the Orbitrap mass analyzer using a resolution of 120,000, and 200 scans were averaged. Replicate spectra for each condition were collected immediately after the initial run.

### Data Processing

Following the acquisition of top-down MS data, deconvolution was performed in Freestyle (v1.7) with Xtract with the analyzer type set to “OT”, the isotope table set to “protein”, and the relative abundance threshold set to 1%. Deconvoluted spectra were then exported from FreeStyle and compared as described in recent work.^44^ Briefly, in-house software extracted up to 100 common peaks with the largest intensities at a mass resolution of 5 ppm. Extracted intensities of fragment ions in the deconvoluted spectra were converted to fractional abundance, and the absolute values of all fractional abundance peaks in the spectra were then taken. These values were then normalized to the average value of the absolute fractional abundance of the fragments from both spectra. Subtracting the normalized absolute fractional abundance for each ion in the spectra yields fractional abundance delta values, quantitative representations of the difference between two ions for a given comparison.

### Statistical Analysis

Fractional abundance deltas for a given condition were compared to those of a replicate average using a two-sample *t* test. The effect size, or Cohen’s d, of each comparison was calculated by subtracting the means of each data set and dividing by the pooled standard deviation. Effect sizes were calculated for all relevant comparisons and then averaged. For example, a comparison between water and 25% methanol was compared to replicates for the water spectrum and to replicates for the 25% methanol spectrum. A post-hoc power analysis was then completed using the TTestIndPower package in Python to determine the power of the comparison assuming an alpha of 0.05 and the calculated effect size. To determine the standard deviation of effect-size measurements used in generating error bars, we split spectra into two 100-scan spectra and analyzed to yield four effect size values.

## Results and Discussion

### Data Processing Pipeline

**Scheme 1:**
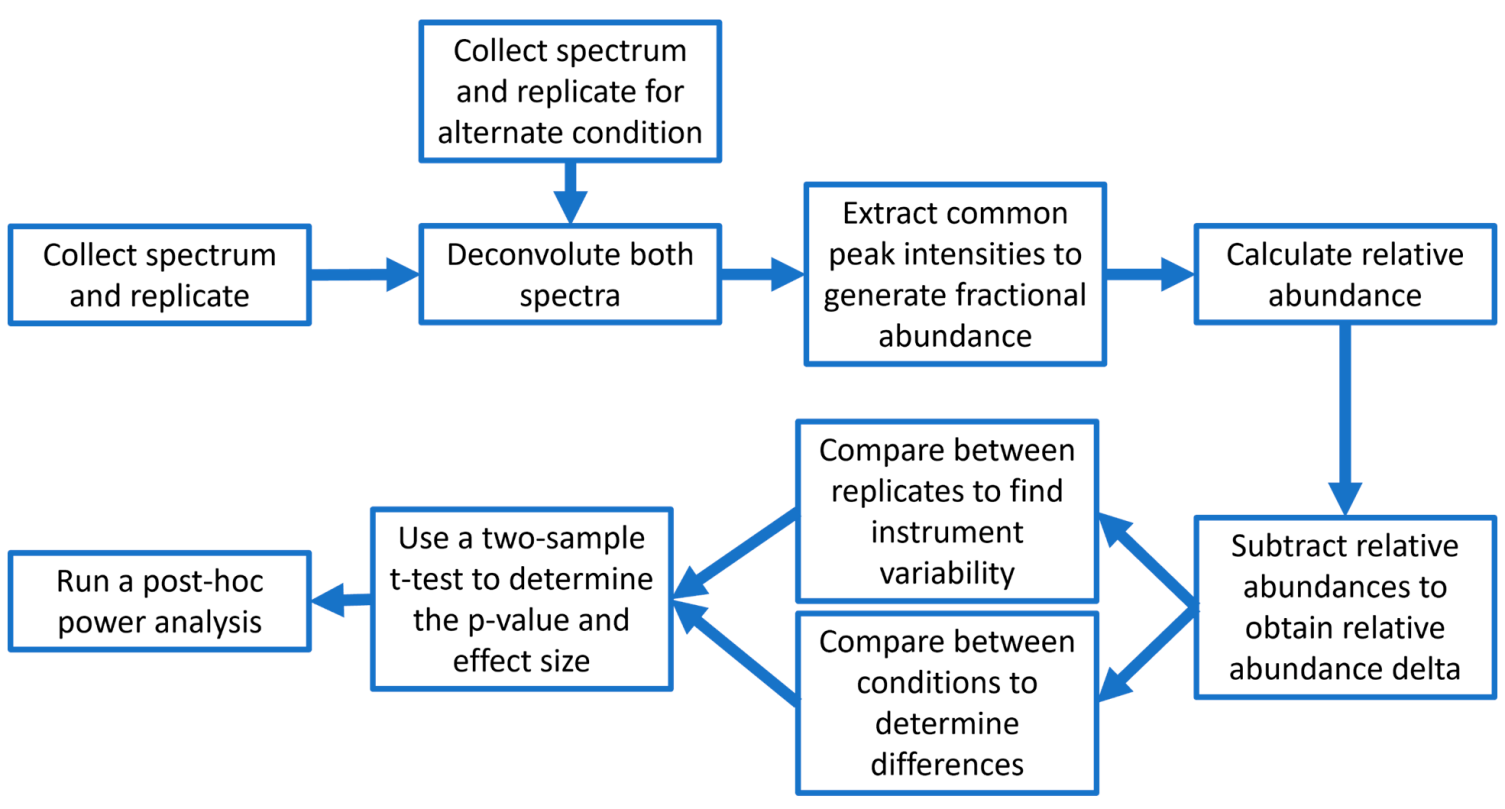
Data Processing Method for Generating Comparisons between Top-Down Spectra

Recently published work from our lab focused on the development of a method to quantify differences in similar mass spectra and provide statistical evaluation of the results.^44^ Briefly, the method relies on changes in the normalized fractional abundance of ions to differentiate between highly similar mass spectra and replicate data. The method is compatible with any dissociation technique and was used to confidently identify peptides with a single site of isomerization. In the present work, we apply the same comparison procedure to assess whether spectra obtained by top-down analysis of intact proteins under a variety of experimental conditions can be confidently distinguished from each other and consider the potential factors that lead to the observed differences. The overall data collection and processing pipeline is illustrated in Scheme 1.

### Thresholds for Statistical Cutoffs

**Figure 1:**
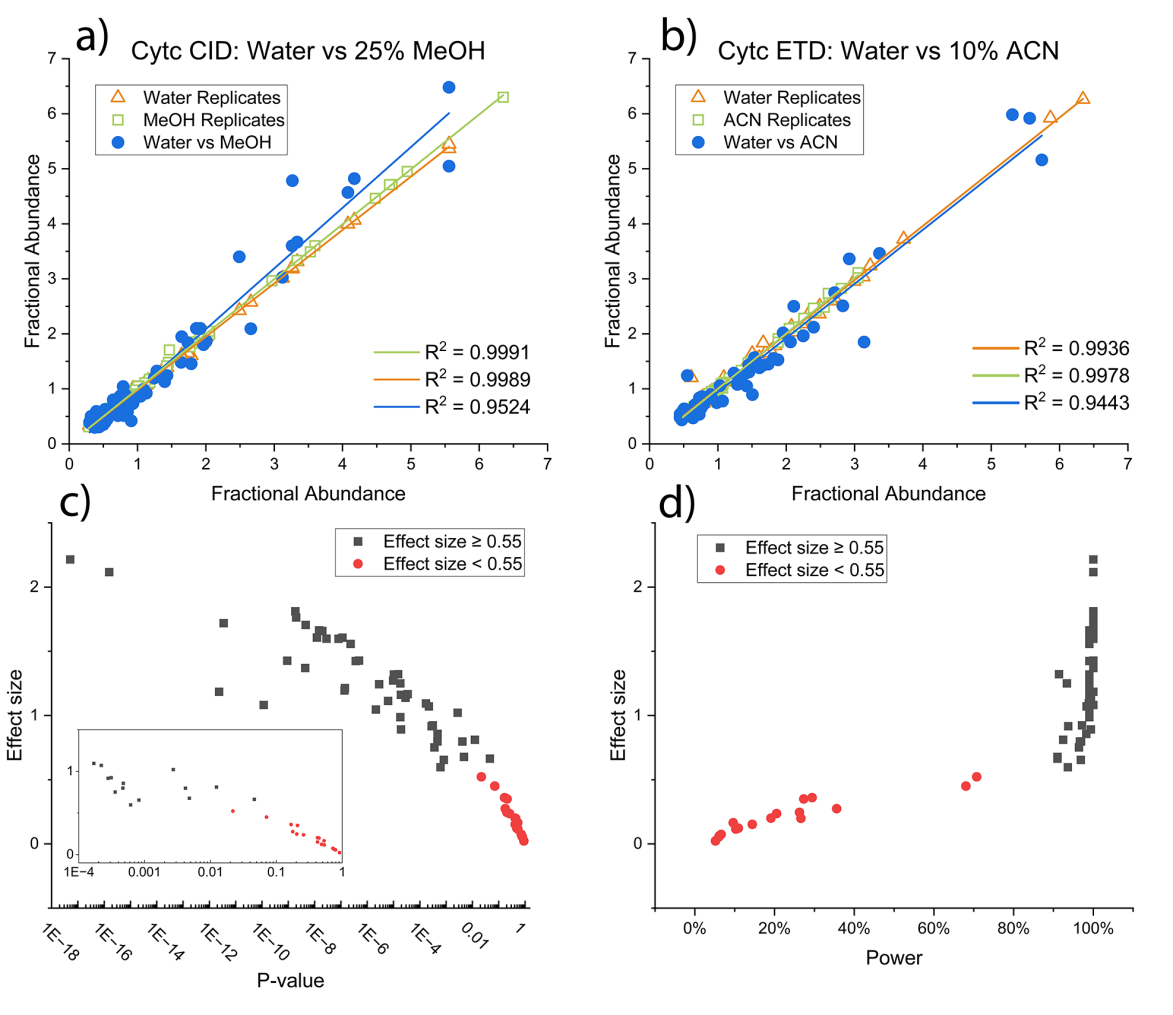
(a,b) Comparisons of fractional abundance of fragment ions between spectra for Cytc in various solvent conditions. (a) Water vs 25% MeOH fragmented by CID; (b) water vs 10% ACN fragmented by ETD. A higher correlation (*R*^2^) between fractional abundances indicates increased similarity between two spectra. (c) Comparison between effect size and *p*-value for all solvent conditions experiments shown in Table 1. *p*-Values are shown on a logarithmic scale. Inset shows the effect sizes for *p*-values between 0.0001 and 1. (d) Comparison between effect size and power for all solvent condition experiments shown in Table 1. For both (c) and (d), any experiment that resulted in fewer than 30 observations was excluded.

To evaluate the reproducibility of potentially subtle changes in top-down MS/MS spectra, we compared replicate results acquired in back-to-back experiments to data acquired from varying solvent conditions (water, water/methanol(75:25), water/acetonitrile (90:10), all with 0.1% formic acid). The results for the 15+ charge state of Cytochrome *c* (Cytc) are shown as scatter plots in Figure 1a and b, for CID and ETD, respectively, under two different solvent conditions. Each data point represents the fractional abundance intersection for ions common to both spectra and above a 1% relative abundance threshold. Results for the replicate spectra are highly correlated in both plots (orange and green data points) and yield a trendline with a slope close to unity. In contrast, the results for comparing fragments from differing solvent conditions yield significantly less correlation (blue data points) by either CID or ETD. To establish whether the differences shown in Figure 1a/b are statistically relevant, we performed many similar experiments and calculated the *p*-value, effect size, and power in each case. P-values are the most used measure of statistical difference between two data sets, with lower *p*-values signifying a higher likelihood that the observed differences are not due to chance. Effect size is a quantitative measure of the differences between two samples based on the differences between their means. Posthoc calculations of statistical power report the likelihood that two samples are different, given the resultant effect size. In Figure 1c and d, effect size versus *p*-value and effect size versus power are plotted, respectively, for each comparison. All of the individual values are also listed in Table 1. Effect sizes greater than 0.55 are generally accompanied by *p*-values below 0.01 and power over 90%, indicating a high likelihood that differences are statistically robust. Therefore, it is possible to use effect size alone to determine statistical reproducibility, and all comparisons yielding an effect size over 0.55 will be considered sufficiently different to reflect statistically sound differences between the reference spectra.

**Table 1:**
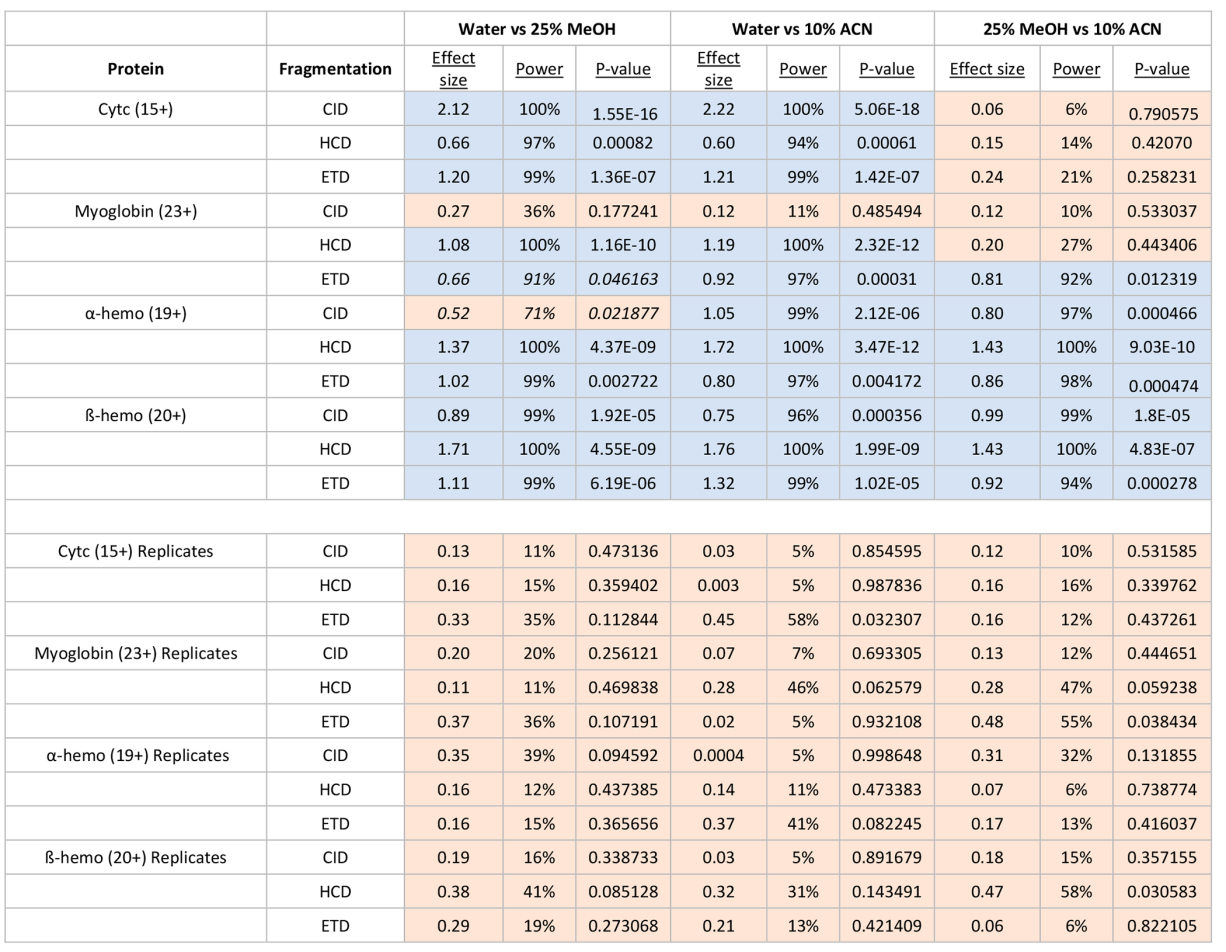
Effect Size, Statistical Power, and *p*-Values for Fractional Abundance Delta Comparisons between Solvent Conditions for Various Proteins^a^

a The charge state of the protein is shown in parentheses next to its name. The top half of the table shows comparisons between the differences in absolute fractional abundance between two conditions compared to those of their respective replicates. Comparisons are highlighted in blue when the observed effect size is above the threshold for statistical difference and in orange when below the threshold (0.55). Italicized values show edge cases in which *p*-values are above 0.01 and have varying results in terms of statistical power. The bottom half of the table shows comparisons between the differences in absolute fractional abundance between two groups of replicates, which would be expected to be smaller than comparisons between two different conditions. Statistical power indicates the likelihood that for the given effect size a *p*-value of 0.05 would reflect differences between the conditions.

**Figure 2:**
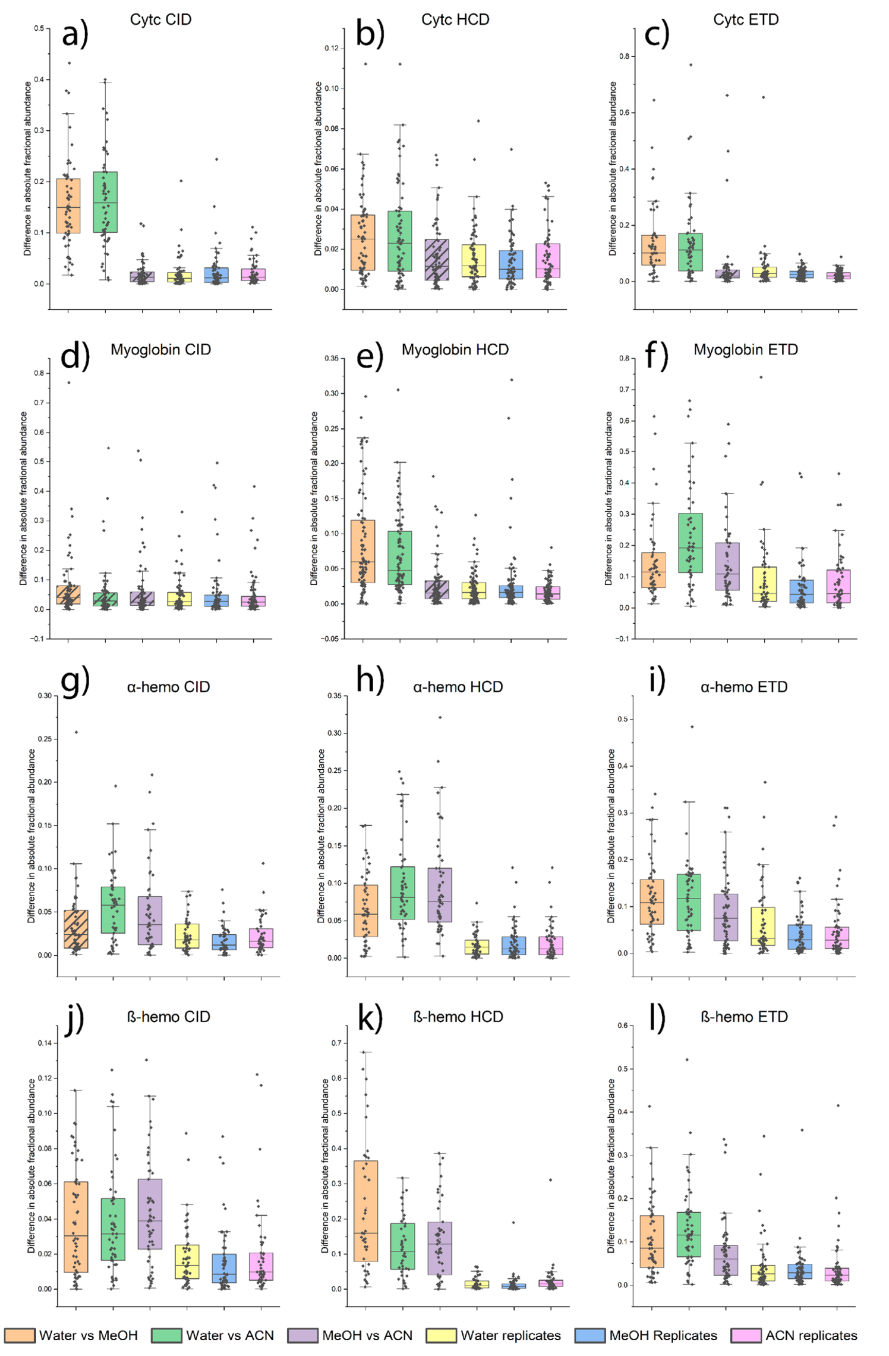
Boxplots of differences in absolute fractional abundance delta when comparing back-to-back spectra to replicate spectra for Cytc (a–c), Myoglobin (d–f), α-hemo (g–i), and β-hemo (j–l). Fragmentation types shown include CID (left column), HCD (middle column), and ETD (right column). Solvent conditions include water (H_2_O), 25% methanol (MeOH), or 10% acetonitrile (ACN). The horizontal line shows the median of the data set. Comparisons that did not produce a significant effect size when compared with replicates are indicated with hatch marks.

With an effect size limit for the significance set, we can now more closely examine the impact of denaturing solvents on the MS-MS spectra for a variety of different proteins. Figure 2 shows box plot summaries of the fractional abundance delta values for each fragment ion as a function of the solvent comparison, protein, and dissociation method. For the 15+ charge state of Cytc, all three dissociation methods are able to distinguish the results obtained in water from those obtained in partial organic solvents. The greatest separation is observed by CID, followed by ETD, and HCD. The results are also consistent for comparisons of MeOH to the ACN solution, which are not distinguishable relative to replicate results by any dissociation method. We also note that the results for the replicates are similar regardless of solvent composition. Although the different electrospray solutions could influence the ionization process, desolvation, or other aspects of the data acquisition, the reproducibility of the replicate results remains nearly constant. This behavior also holds true regardless of the target protein, as seen in the remainder of Figure 2. The results for the 23+ charge state of myoglobin differ from those obtained for Cytc. CID is unable to distinguish any of the solvent conditions, which may be related to the presence of favored cleavages at Asp/Pro residues. In contrast, HCD can distinguish between water and both mixed water/organic solvents, while ETD is able to differentiate all solvent conditions. For α-hemoglobin (α-hemo) and β-hemoglobin (β-hemo) (19+ and 20+ charge states, respectively), all experiments yield statistically discernible differences with the exception of CID for α-hemo water vs partial MeOH.

In general, ETD is able to distinguish spectra from the greatest number of solvent conditions, although the magnitudes of differences for ETD are typically smaller than those for HCD/CID (when those methods are successful). Given that statistically differentiable fragmentation spectra are produced for a variety of proteins for which only the electrospray solution was changed, it is worth considering the factors potentially contributing to these observations. Changes in the solvent composition may alter the protein structure ultimately produced in the gas phase. These differences could manifest in either the three-dimensional orientation of the backbone and side chains or in the protonation/deprotonation sites (or in differences in both). Previous results suggest that ETD may be sensitive to proton localization. Work from Zubarev and co-workers in 2006 used electron-based fragmentation to determine the location of charge sites in gas-phase polypeptide cations based on preferential neutralization of protons located at basic sites.^45^ With these results in mind, we can infer that methanol and acetonitrile may impact the localization of charges in myoglobin, which is also supported by other previous work. For example, tyrosine electrosprayed from methanol produces a mixture of phenoxide and carboxylate ions, while from acetonitrile it produces primarily the carboxylate ion.^46^

The fact that CID can distinguish between solvent conditions is perhaps most surprising. CID occurs after slow heating of the ion by many collisions, which might suggest that regardless of differences in structure, annealing during the slow heating might erase any memory of those differences. In some cases, this may in fact happen, but our positive results suggest that some differences in either structure or charge localization are separated by barriers greater than those leading to fragmentation, in agreement with work discussed above.^36^ Relative to CID which activates ions over milliseconds, HCD fragmentation takes place on a shorter time scale (μs) following a smaller number of more energetic collisions.^47^ The observation that HCD tends to produce more observable fragments than CID suggests that HCD affords access to higher energy dissociation pathways or sequential fragmentation events. Arguments can be made for these additional pathways either favoring or disfavoring retention of structure, which makes it difficult to pinpoint why differences for some proteins vary between HCD and CID. Regardless of the precise underlying causes, our results suggest that some combination of charge localization and three-dimensional structure lead to discernible differences in MS/MS spectra.

**Figure 3:**
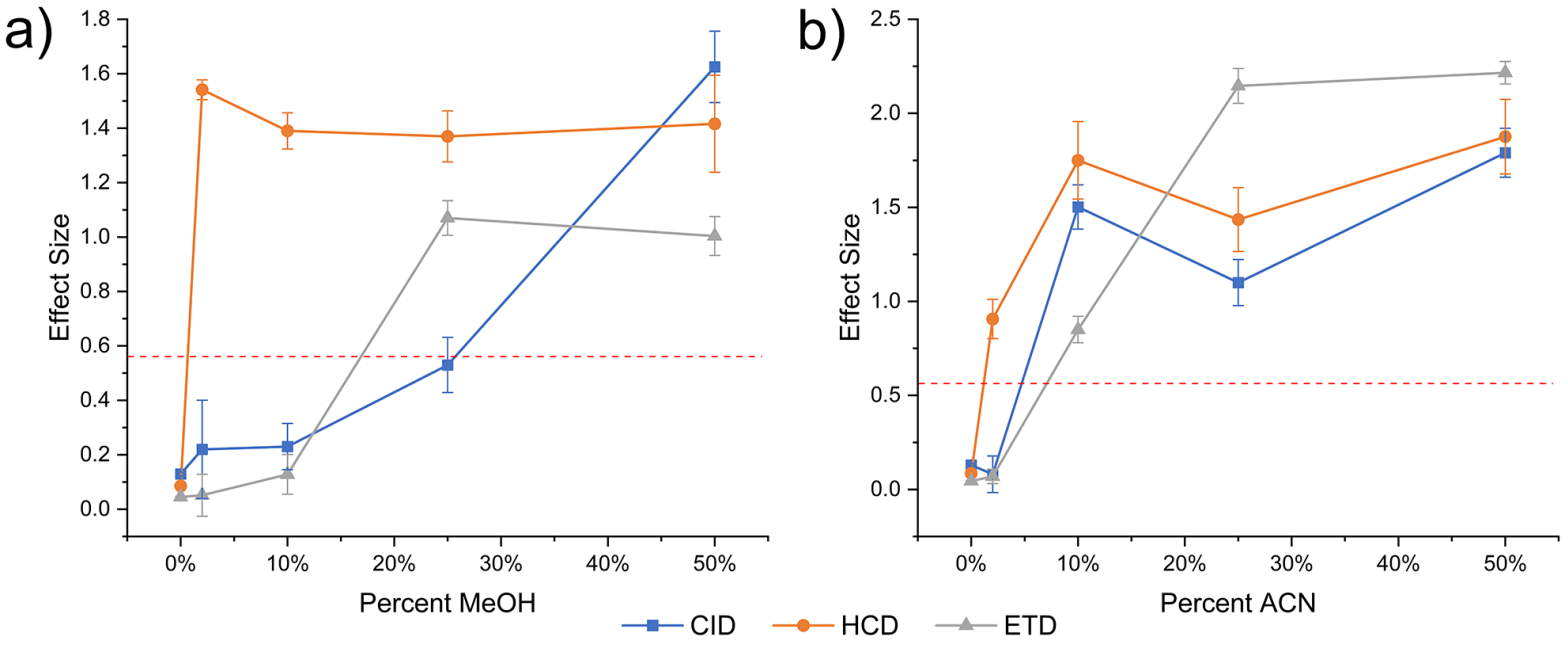
Effect size dependence on solvent composition for comparisons against water for α-hemo. The dashed red line represents the minimum effect size for two comparisons to be considered significantly different. (a) Percentage of MeOH in solvent and the resultant effect size of the comparison against water. (b) Percentage of ACN in solvent and the resultant effect size of the comparison against water.

To further explore the effect of solvent conditions, we varied the amounts of MeOH and ACN while monitoring the fragmentation of 19+ α-hemo. The results are summarized in Figure 3. For HCD, addition of 2%, 10%, 25%, and 50% MeOH all yield similar effect sizes well above the threshold for differentiation. Although the effect sizes of these comparisons are similar, examining the details of the underlying delta values provides more information. As the difference in the amount of denaturant between any two conditions increases, the correlation between delta values decreases. This suggests that as the amount of denaturant changes, the contributions of specific ions vary, but the overall extent of differences in fractional abundance remains the same. In contrast, CID is only able to distinguish data from the 50% MeOH sample, although the effect size is large for that experiment. For ETD, results obtained for 25% and 50% MeOH yield similar effect sizes around ∼1. Varying the ACN % revealed different trends for each dissociation method compared to MeOH. Although 2% ACN yielded a significant effect size for HCD, subsequently, higher percentages of ACN yielded larger effect sizes. For CID, 2% yielded no effect, but 10%, 25%, and 50% each yielded consecutively higher effect sizes, consistent with additional changes to fragmentation with the additional organic component. For ETD, a small effect was observed at 10%, which grew significantly at 25% and remained similar at 50%. The varying trends in Figure 3 suggest that changes in the protein that are detectable by one dissociation method may not be discernible by another. Furthermore, it is clear that identical amounts of MeOH or ACN influence protein structure or charge localization in different ways.

### Subthreshold Collisional Activation

**Figure 4:**
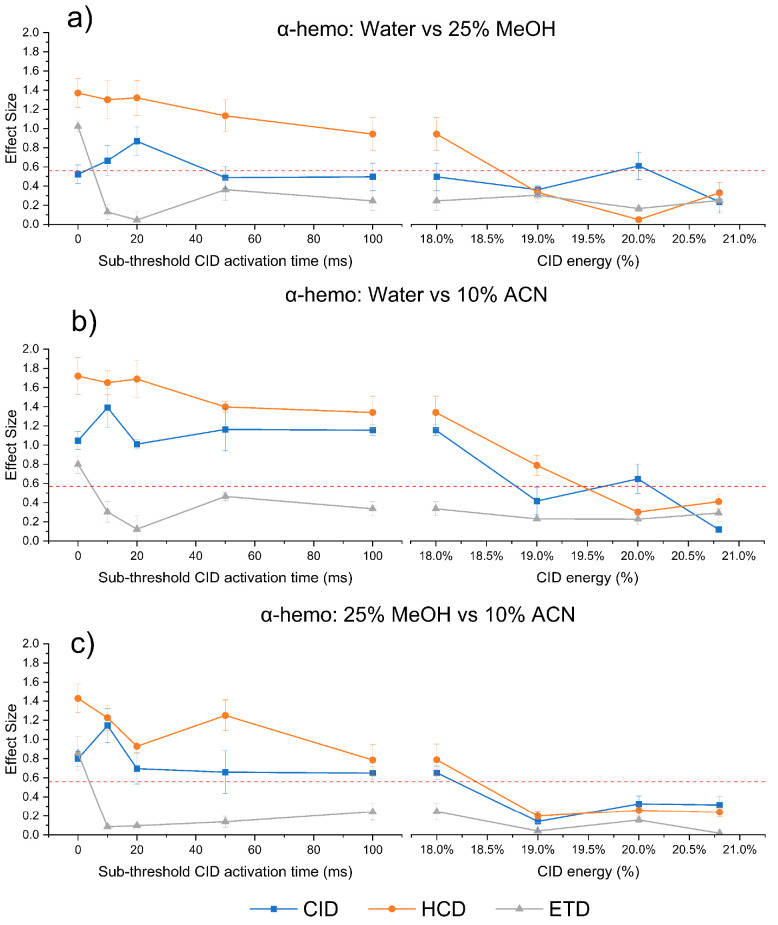
Application of subthreshold CID reduces structural differences via annealing prior to fragmentation. Impact of subthreshold CID activation time (left) and CID energy (right) when comparing between water and 25% MeOH (a), water and 10% ACN (b), and 25% MeOH and 10% ACN (c). Plots where CID energy is increased beyond the fragmentation threshold (right) are at a constant activation time of 100 ms. The red dashed line shows the minimum effect size for statistically relevant differences.

To further explore whether metastable structural features formed during the electrospray process or residual structural elements of the native state account for the differences in dissociation that we observe, subthreshold collisional activation experiments were conducted to anneal proteins toward the lowest-energy gas-phase structure. The application of collisional energy through resonant excitation in an ion-trap slowly heats ions in the gas phase, potentially enabling unfolding and structural rearrangement in the case of proteins. By applying collisional energy below the threshold for fragmentation, additional annealing time and energy can be applied to the ions.^48,49^ We repeated comparisons between back-to-back experiments and varying solvent conditions with subthreshold collisional activation applied in MS^2^. The collision energy applied in MS^2^ was selected by incrementally increasing the excitation until small fragments began to form, followed by reduction of the energy just below this level. The detailed outcomes of these experiments are provided in Table S1. For all statistically significant comparisons shown in Table 1, subthreshold CID leads to a decrease in the differences in fractional abundance and resulting effect sizes. However, α-hemo and β-hemo show more modest reductions in effect size compared to other proteins, with many comparisons retaining statistically significant effect sizes after the supplemental activation. To further investigate the impacts of annealing on α-hemo, we also increased the collisional activation time as shown in the left panels of Figure 4.

For HCD-type MS^3^ fragmentation (orange), increasing annealing time decreases the effect size for all solvent conditions. This suggests that longer activation time allows for more annealing and a shift toward a common gas-phase structure. Fragmentation by CID (blue) appears to cause an initial increase in effect size at shorter annealing times followed by a return to nearly the original effect sizes at longer activation times. Although annealing should lead to a common-gas phase structure eventually, these results suggest that disparate intermediate conformations may be accessed and detected before coalescence to a common structure occurs. When subthreshold activation is applied prior to ETD (gray), the effect size of comparisons rapidly decreases below the threshold for significance for all solvent conditions. This reveals that differences in ETD derive from structural features that are easily erased by mild collisional activation. One interpretation of this observation is that proton/salt bridge localization is primarily responsible for differences in ETD, and that these sites are rapidly scrambled due to increased proton mobility during mild heating. These results also imply that, at least for α-hemo, ETD may not be particularly sensitive to three-dimensional structure (given the differing behavior relative to HCD and CID). Furthermore, when assigning ETD fragment ions for Cytc, the fragments with the largest changes in fractional abundance are located on or adjacent to basic amino acids, potential charge sites (Figure S4). Taken together, these results suggest that ETD mainly detects changes in charge localization.

Although increasing annealing times often led to a decrease in the effect size, several solvent conditions retained high enough effect sizes to be considered statistically significant after considerable activation. Furthermore, the data appeared to plateau for the HCD and CID comparisons of ACN/H_2_O, suggesting that additional time may not be sufficient to overcome barriers between structural states. To provide additional energy toward annealing, we increased collisional activation beyond the threshold for fragmentation of the protein, while retaining the 100 ms activation time, and then reisolated the unfragmented population to be fragmented in MS^3^. While this does result in some decrease in signal, additional heating of the ions should be attained. As shown in the right panels of Figure 4, the effect sizes eventually decrease below the threshold for statistical significance in all cases. These results suggest that with sufficient activation, α-hemo can be annealed to a uniform structure in the gas phase from a variety of disparate initial structures.

### Proton Transfer Charge Reduction

**Scheme 2:**
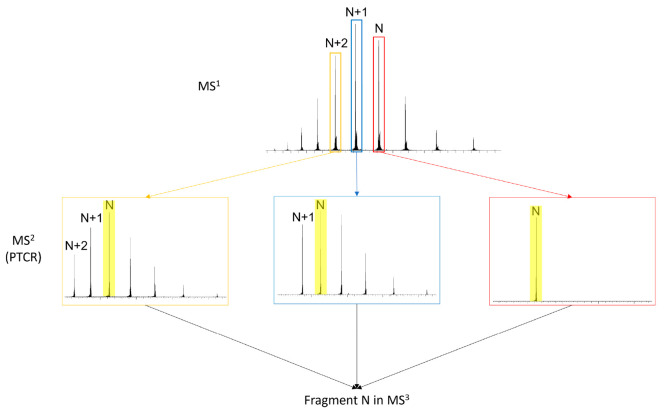
Experimental Design of Proton Transfer Charge Reduction (PTCR) Experiments The N charge state is isolated in MS^2^ without PTCR (red) and is fragmented in MS^3^. The N+1 and N+2 charge states are isolated in MS^2^ and undergo PTCR reactions to create reduced charge state distributions (yellow and blue). The N charge state from those distributions is then isolated in MS^3^ and fragmented.

To further explore the potential effects of structural rearrangement in the gas phase, we examined differences in fragmentation following proton transfer charge reduction (PTCR) reactions. PTCR can be used to decrease the charge state of a multiply protonated ion through ion–ion reactions with a suitable anion.^43^ These reactions have been shown to reduce Coulombic interactions and yield compact structures,^24^ but these compacted structures may not resemble native-like structures preserved from solution.^50^ To probe whether charge state reduction by PTCR can lead to detectable changes in protein structures of the same final charge state, we applied PTCR prior to fragmentation and analyzed the fractional abundance differences in fragment ions. As illustrated in Scheme 2, we chose a charge state to fragment (N) and then used proton transfer to charge-reduce the two next higher charge states (N+1, N+2) down to the same final charge state. The results of these experiments are shown in Table 2.

**Table 2:**
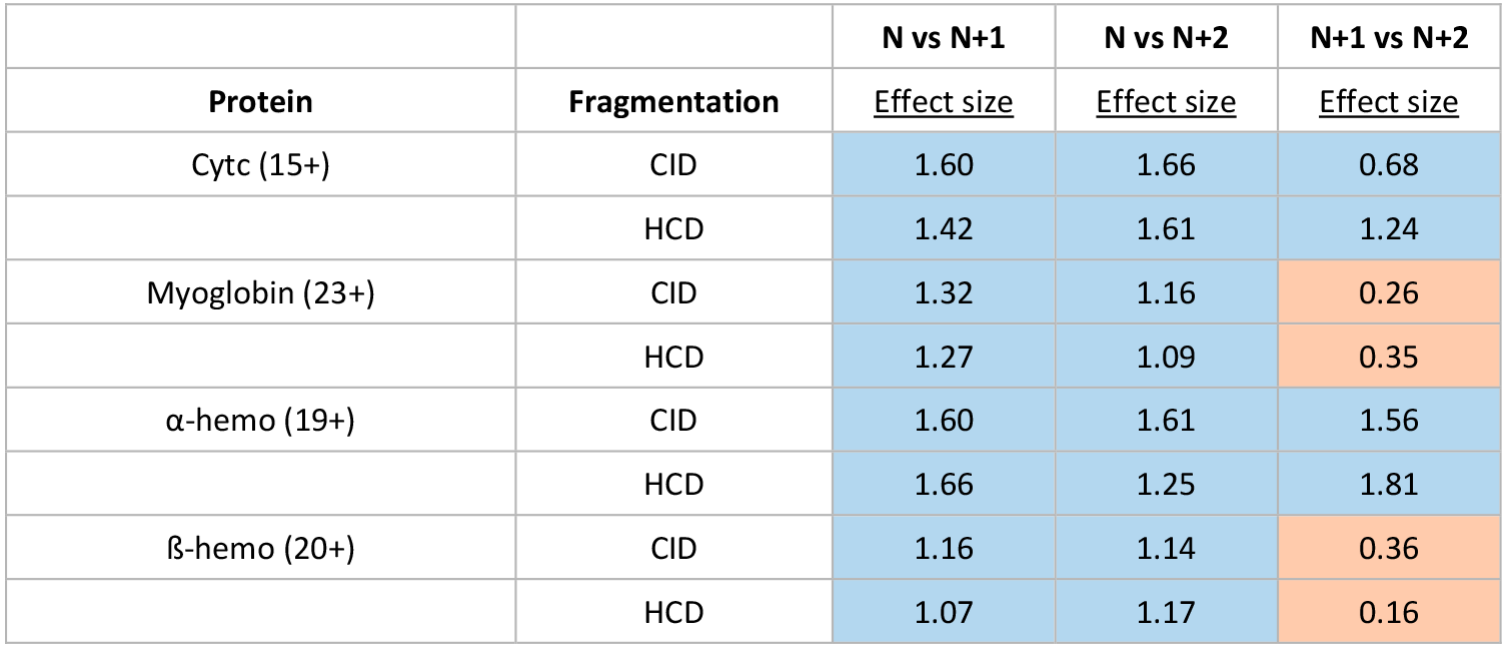
Effect Size for Comparisons between the Normalized Fractional Abundance of Fragment Ions between PTCR Experiments^a^

a The fragmented charge state of the protein (N) is shown in parentheses next to the name of the protein. Comparisons are highlighted blue when the observed effect size is above the threshold for statistical difference and orange when below the threshold (0.55).

For all proteins and all dissociation methods, the comparison of the original ion to both the singly and doubly charge-reduced versions yielded large effect sizes. This suggests that for proteins, charge reduction does not in general yield structures similar to those generated directly by electrospray. Given that PTCR does not remove protons in a selective fashion (meaning that any proton can be removed) and that the removal of any given proton could influence the subsequent structure that is observed differently, perhaps the results in Table 2 are not surprising. Further information can be obtained by comparison of the N+1 and N+2 forms, which yield effect sizes below 0.55 for both myoglobin and β-hemo. This observation is consistent with significant scrambling of the system into many structural forms (rather than changes in proton location) following nonselective proton removal, preventing differentiation of the results. However, for the remainder of the proteins, higher effect sizes were observed including values well over 1. For these proteins, proton removal may not be stochastic due to structural features that shield some protons from abstraction. Indeed, examination starting with the 24+ charge state for myoglobin yields higher effect sizes (Table S2), suggesting that proton accessibility may change as a function of charge state/structure. Overall, these results show that PTCR reactions can change the structure and charge localization of protein ions, and further confirm that the structure of a protein in the gas phase (even for the exact same charge state) can be dependent on the conditions under which it was created.

## Conclusion

Our results illustrate that differences in the normalized fractional abundance of fragment ions in top-down mass spectra are correlated with subtle changes in the tertiary protein structure and charge localization. Using quantitative statistics, small but significant perturbations in protein structure can be detected, and the method allows for comparison between any two conditions of interest. Furthermore, the unique properties of the fragmentation methods can be used to provide additional information. For example, CID and HCD analyses are likely to report differences in three-dimensional protein structure, whereas differences in ETD appear to be more derived from changes in charge localization. Attenuation of fractional abundance differences in the presence of subthreshold collisional activation further affirms that protein structure and charge localization are encoded in fragment-ion abundance and that structures can be subject to change in the gas phase. We observed that even small amounts of denaturants can influence the structure or charge localization of model proteins, highlighting that a high degree of care must be taken during sample preparation if structural comparisons are to be made. Changes induced *in vacuo* by ion–ion reactions can also exert a strong influence on gas-phase structure and charge localization. When executed carefully, comparison between the fractional abundances of fragment ions in tandem top-down mass spectra is an accessible, versatile tool for protein structure analysis.

## Supporting Information Available

Representative CID, HCD, and ETD spectra, impact of increasing denaturant on hemoglobin spectra, 10 ms subthreshold CID effect sizes, assignments for Cytc ETC spectra, myoglobin PTCR effect sizes (PDF)
