# Supplementary material for: Fragment Ion Abundance Reveals Information about Structure and Charge Localization in Highly Charged Proteins
Source: J Am Soc Mass Spectrom. 2023 Jul 21;34(8):1778–88. doi: 10.1021/jasms.3c00196 (PMC10401701; doi:10.1021/jasms.3c00196)
Supplement: Supplementary file 1 — js3c00196_si_001.pdf [file js3c00196_si_001.pdf]

Supporting Information For:

Fragment ion abundance reveals information about structure and charge localization in highly charged proteins

Thomas A. Shoff and Ryan R. Julian*

Department of Chemistry, University of California, Riverside, California 92521, United States

* Corresponding author: Ryan R. Julian
E-mail: ryan.julian@ucr.edu

Keywords: native mass spectrometry, top-down mass spectrometry, collisional activation, charge localization, electron-transfer dissociation

Figure S1: Representative CID, HCD, and ETD spectra for each protein. (a) Cytc CID (b) Cytc HCD (c) Cytc ETD (d) Myoglobin CID (e) Myoglobin HCD (f) Myoglobin ETD (g) α-hemo CID (h) α-hemo HCD (i) α-hemo ETD (j) ß-hemo CID (k) ß-hemo HCD (l) ß-hemo ETD

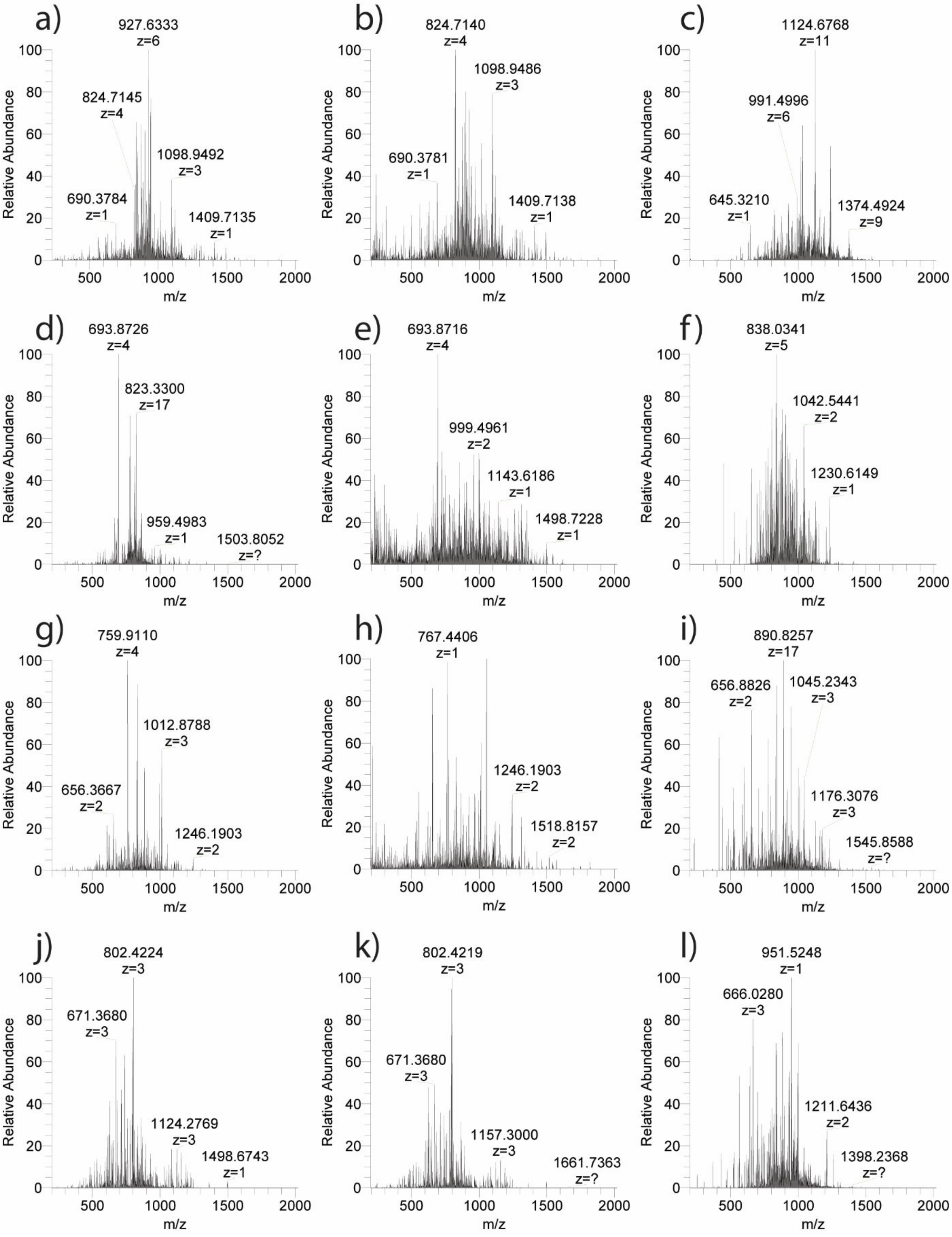

Figure S2: Impact of increasing acetonitrile concentration on charge state distribution of hemoglobin alpha chain.

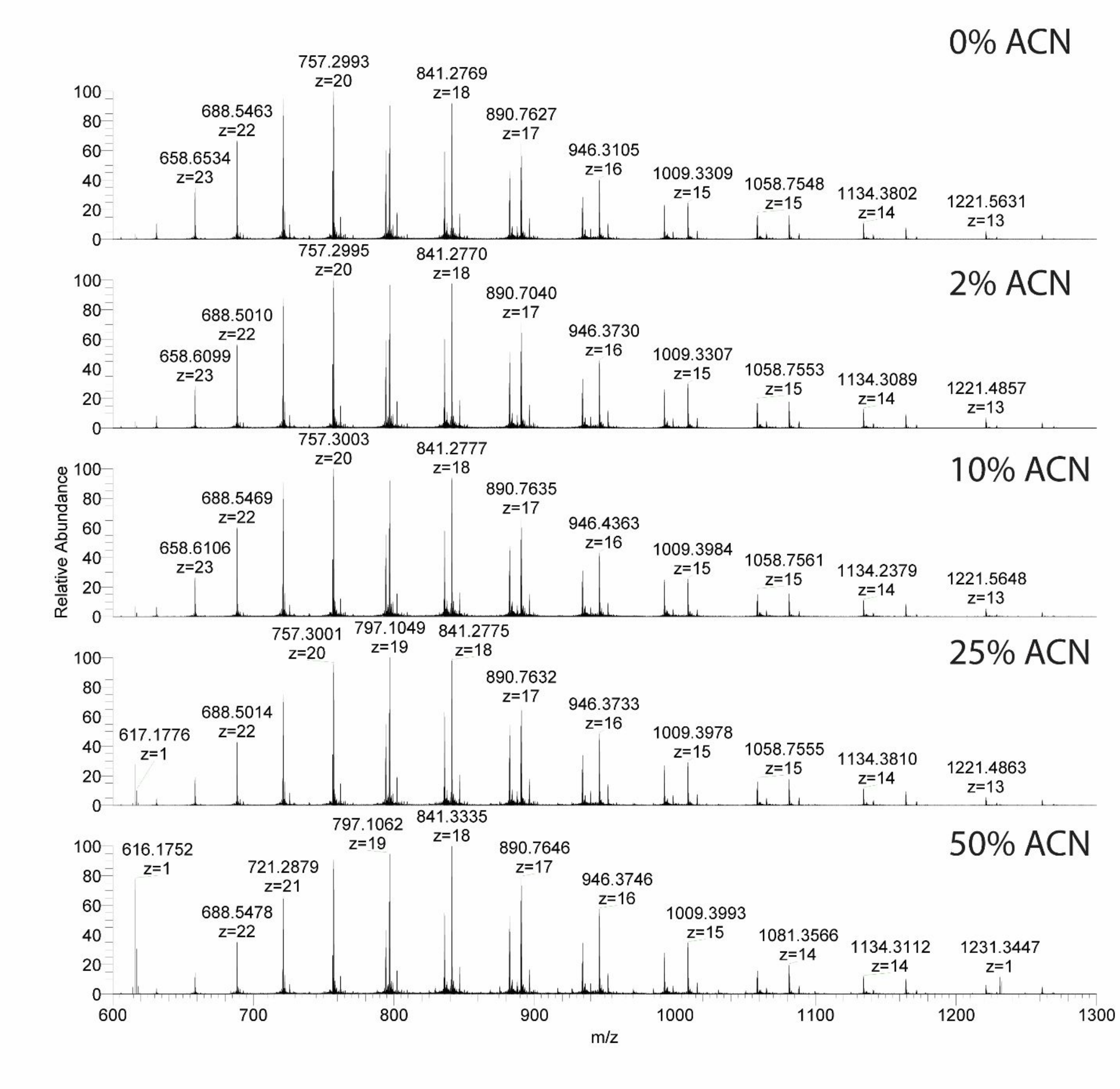

Figure S3: Impact of increasing methanol concentration on charge state distribution of hemoglobin.

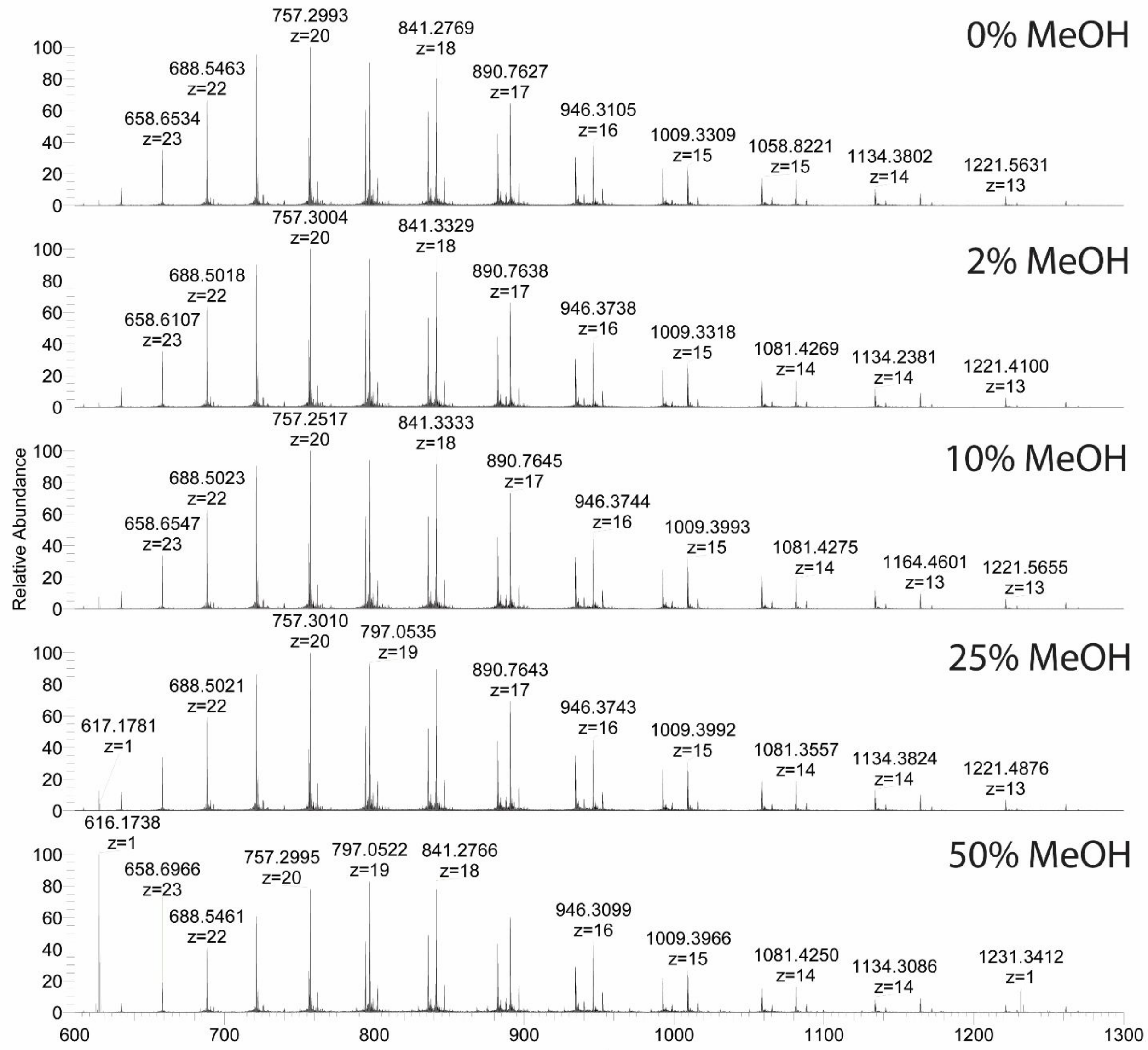

Table S1: Effect size for relative abundance delta comparisons between solvent conditions for various proteins following 10 ms sub-threshold collisional activation. The charge state of the protein is shown in parentheses next to its name.

		**Water vs 25% MeOH**	**Water vs 10% ACN**	**25% MeOH vs 10% ACN**
**Protein**	**Fragmentation**	Effect size	Effect size	Effect size
Cytc (15+)	CID	0.70	0.61	0.21
	HCD	0.19	0.07	0.18
	ETD	0.11	0.14	0.09
Myoglobin (23+)	CID	0.22	0.20	0.20
	HCD	0.13	0.03	0.12
	ETD	0.28	0.32	0.08
α-hemo (19+)	CID	0.66	1.39	1.14
	HCD	1.30	1.65	1.23
	ETD	0.13	0.30	0.09
ß-hemo (20+)	CID	0.41	0.49	0.10
	HCD	0.94	1.12	0.85
	ETD	0.03	0.32	0.07

Figure S4: Assignments of the ions with the largest differences in fractional abundance for Cytc fragmented by ETD. (a) Water vs 25% MeOH (b) Water vs 10% ACN

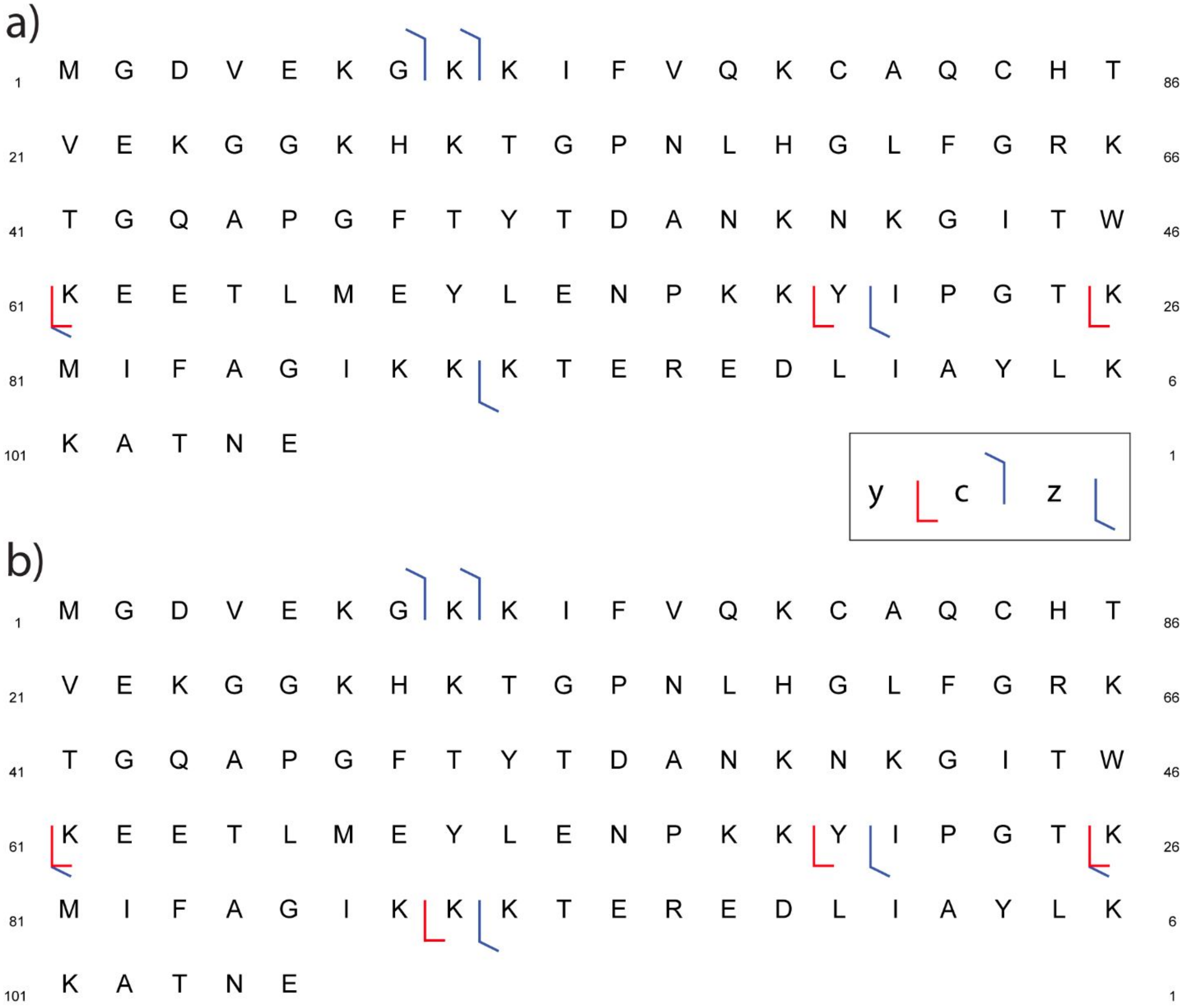

Table S2: Effect size for relative abundance delta comparisons between proton transfer charge reduction experiments for Myoglobin in the 24+ charge state. Statistical power indicates the likelihood that for a given effect size, a p-value of 0.05 would reflect differences between conditions.

		**N vs N+1**	**N vs N+2**	**N+1 vs N+2**
**Protein**	**Fragmentation**	Effect size	Effect size	Effect size
Myoglobin (23+)	CID	1.32	1.16	0.26
	HCD	1.27	1.09	0.35
Myoglobin (24+)	CID	n/a	n/a	0.54
	HCD	n/a	n/a	1.26
